# Structural Reorganisation and Potential Toxicity of Oligomeric Species Formed during the Assembly of Amyloid Fibrils

**DOI:** 10.1371/journal.pcbi.0030173

**Published:** 2007-09-14

**Authors:** Mookyung Cheon, Iksoo Chang, Sandipan Mohanty, Leila M Luheshi, Christopher M Dobson, Michele Vendruscolo, Giorgio Favrin

**Affiliations:** 1 Department of Chemistry, University of Cambridge, Cambridge, United Kingdom; 2 Computational Proteomics and Biophysics Laboratory, Department of Physics, Pusan National University, Busan, Korea; 3 John von Neumann-Institut für Computing, Forschungszentrum, Jülich, Germany; Harvard University, United States of America

## Abstract

Increasing evidence indicates that oligomeric protein assemblies may represent the molecular species responsible for cytotoxicity in a range of neurological disorders including Alzheimer and Parkinson diseases. We use all-atom computer simulations to reveal that the process of oligomerization can be divided into two steps. The first is characterised by a hydrophobic coalescence resulting in the formation of molten oligomers in which hydrophobic residues are sequestered away from the solvent. In the second step, the oligomers undergo a process of reorganisation driven by interchain hydrogen bonding interactions that induce the formation of β sheet rich assemblies in which hydrophobic groups can become exposed. Our results show that the process of aggregation into either ordered or amorphous species is largely determined by a competition between the hydrophobicity of the amino acid sequence and the tendency of polypeptide chains to form arrays of hydrogen bonds. We discuss how the increase in solvent-exposed hydrophobic surface resulting from such a competition offers an explanation for recent observations concerning the cytotoxicity of oligomeric species formed prior to mature amyloid fibrils.

## Author Summary

Several peptides and proteins have been shown to convert from their soluble forms into highly ordered fibrillar aggregates, known as amyloid fibrils. It has also been realised that the formation of amyloid fibrils is often preceded by the appearance of small but highly organised oligomeric assemblies. Interest in these low molecular weight oligomers has increased considerably since these molecular species have been detected in the brains of patients suffering from Alzheimer disease. Evidence is accumulating concerning the ability of the low molecular weight oligomers formed by the Aβ peptide to specifically disrupt cognitive function. To increase our understanding of this phenomenon, we describe in this paper the early stages of the oligomerization process of two fragments (Aβ_16–22_ and Aβ_25–35_) of the Aβ peptide, by exploiting the possibility provided by computer simulations to describe aggregation reactions at the molecular level. Our results suggest that the ability of many diverse peptides and proteins to form amyloid fibrils, as well as the inherent toxicity of many oligomeric assemblies, are a consequence of the tendency of the backbone of polypeptide chains to form hydrogen bonds, and of the outcome of the competition between hydrophobic and hydrogen bonding forces.

## Introduction

The phenomenon of protein aggregation is associated with a variety of human disorders [1–3], which include Alzheimer and Parkinson diseases, type II diabetes, and the spongiform encephalopathies, that are rapidly becoming a major challenge in terms of their social and economic impact on society. In addition, there is increasing evidence that the formation of ordered aggregates, known as amyloid fibrils, is not just an unusual characteristic of the 20 or so proteins associated with disease, but rather is just one of the possible conformations that any polypeptide chain can adopt under appropriate conditions [4–6]. Questions of great scientific and medical interest in this context include the molecular basis of amyloid formation and the nature of the toxic species that appear to be associated with amyloid formation in vivo and to result in pathogenic cell death in at least some of these diseases [6].

Experimental studies have shown for a range of peptides and proteins that amyloid fibril formation is preceded by the appearance of organised molecular assemblies usually termed protofibrils [7–11]. In addition, detailed biophysical studies are beginning to identify the formation of smaller oligomeric species at yet earlier stages of the aggregation process [12,13]. These oligomers appear initially to be relatively disordered, but then to convert into species containing extensive β sheet structure [10] that are often capable of stimulating fibril formation [14]. Interest in these low molecular weight oligomers has increased since these species have been detected in the brains of patients suffering from Alzheimer disease [15,16]. Although the identification of the species giving rise to neurodegeneration is one of the most controversial topics in current studies of misfolding diseases, evidence is accumulating concerning the ability of the low molecular weight oligomers of the Aβ peptide specifically to disrupt cognitive function [17–21].

Even if highly complex processes are associated with oligomer toxicity, a view is gaining support according to which the ability to form toxic oligomeric species represents an intrinsic property of polypeptide chains at some stage of their oligomerization process [22–26]. In addition, a link has been made recently between the toxicity and the hydrophobicity of polypeptide chains [27–29]. Despite this growing interest in the role of peptide and protein oligomers in disease, the molecular mechanism by which they are formed remains to be fully elucidated. According to the “nucleated conformational conversion” (NCC) model for aggregate formation [14], a group of monomers initially present in solution coalesces to form “molten” oligomers, which subsequently undergo a reorganisation process and eventually give rise to more highly organised oligomers and fibrils rich in β sheet structure.

Although the nucleated conformational conversion mechanism has been supported by many experimental and theoretical observations [11,14,30–34], a detailed description of this process at the molecular level remains in large part elusive because it is challenging to describe the early stages of aggregation of polypeptide chains by experiment, primarily because of the difficulties in detecting and characterising the small, structurally heterogeneous, and transient species that are involved.

The mechanism by which structured oligomers might grow upon addition of unstructured monomers has, however, been explored in a variety of computer simulations [34,35]. One particular type of mechanism, usually called dock and lock, was initially proposed by Esler et al. [36] to explain the experimental observation of the growth of Aβ fibrils. According to this idea, the attachment of a monomer to a preformed ordered oligomer or fibril is followed by a much slower and rate-limiting lock phase. Recent simulations [35] suggest that in this latter step the preformed oligomer undergoes a series of conformational fluctuations in order to accommodate the new peptide molecule.

In this paper we investigate the early stages of the oligomerization process for two fragments (Aβ_16–22_ and Aβ_25–35_) of the Aβ peptide, a key pathogenic agent in Alzheimer disease [21] by using all-atom computer simulations (see Methods) able to describe the essence of peptide aggregation reactions at the molecular level [33–35, 37–48]. 

Our results suggest that amyloid fibril formation is a generic property of protein aggregation [4–6] as a consequence of the fact that the ability of the main chain to form hydrogen bonds is common to all polypeptide chains and that the competition between hydrophobicity and hydrogen bonding is a major determinant of the aggregation process. They also suggest that the mechanism of aggregation that leads to the structural reorganisation involved in the growth of ordered oligomeric species could be a key determinant of their toxic properties.

## Results

### Simulations and Their Analysis

To study in detail the formation of large oligomers, we carried out two series of 100 independent all-atom simulations using the ProFASi (Protein Folding and Aggregation Simulator) program [49–53] (see Methods) for systems of 20 peptides for each of the two fragments, Aβ_16–22_ (KLVFFAE) and Aβ_25–35_ (GSNKGAIIGLM).

**Figure 1:**
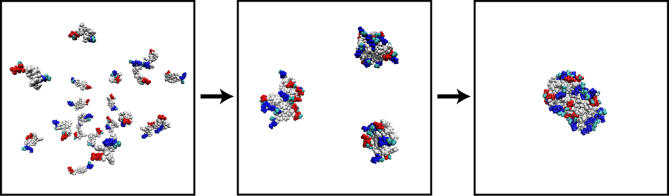
Representative Conformations in a Monte Carlo Simulation Representative steps in a Monte Carlo simulation of Aβ_16–22_ showing the coalescence of individual peptide molecules into aggregates.

Under the conditions that we used here, the peptides were observed to interact strongly with each other and to form oligomers (Figure 1), which are described as an assembly of *N_c_* strongly interacting peptides. Peptides are considered to be interacting when the sum of their interchain hydrogen bonds and hydrophobic interactions is greater than a cutoff value of 1.5 in model units, which corresponds approximately to the formation of two hydrogen bonds. According to this definition, oligomers may be found in many different conformations ranging from disorganised molten assemblies to well-ordered arrays of molecules rich in β sheet structure.

To follow their assembly process in more detail, we further distinguish between ordered β sheet rich regions and disordered amorphous ones by introducing a local-order parameter β that is calculated at the individual residue level and then averaged over the entire ensemble of 20 peptides (see Methods). If all residues of all peptides are in a β sheet configuration, β = 1, and if no residues of any molecule are in such structure, β = 0. To probe the early stages of the process associated with the formation and growth of low molecular weight oligomers, we also measure the time evolution of properties of single oligomers, rather than of the ensemble of all peptides. For this reason, we define a local-order parameter *β_o_*, which is averaged only over the peptides that are contained in one specific oligomer.

To focus on the assembly and structural formation of these oligomers that are likely to involve substantial transitions as the size of oligomers increases, we analysed a range of temperatures and concentrations to identify the appropriate conditions (see Methods). On the basis of these studies, we chose to study the aggregation of both Aβ_16–22_ and Aβ_25–35_ inside a cubic box with periodic boundary conditions of (60 Å)^3^ and a temperature of 295 K.

### Time Evolution of the Populations of Oligomers

**Figure 2:**
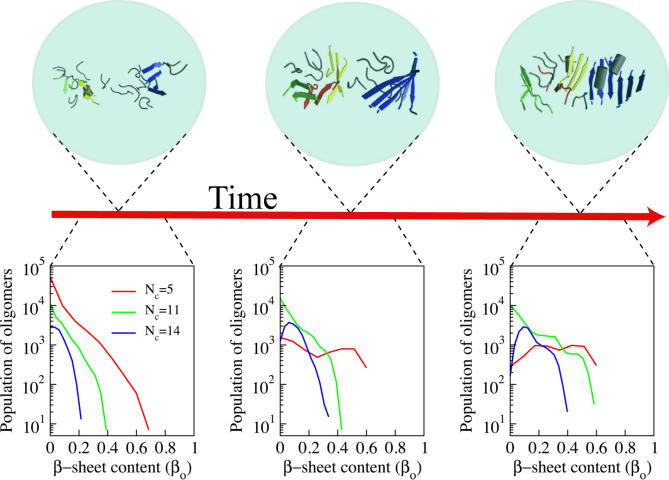
Time Dependence of the Population of Aβ_16–22_ Oligomers The population *P*(*N_c_*) was calculated as the sum over all the oligomers of a given size (*N_c_* = 5 (red), 11 (green), and 14 (blue)) in a specific time window and in all the 100 independent simulations. We show *P* as a function of the β sheet content as defined by the local-order parameter β_o_. Each window corresponds to a different time point along the simulation. At early stages (left), configurations are highly disordered; at intermediate stages (centre), ordered structures start to appear; at late stages (right), the structures are rather well ordered.

**Figure 3:**
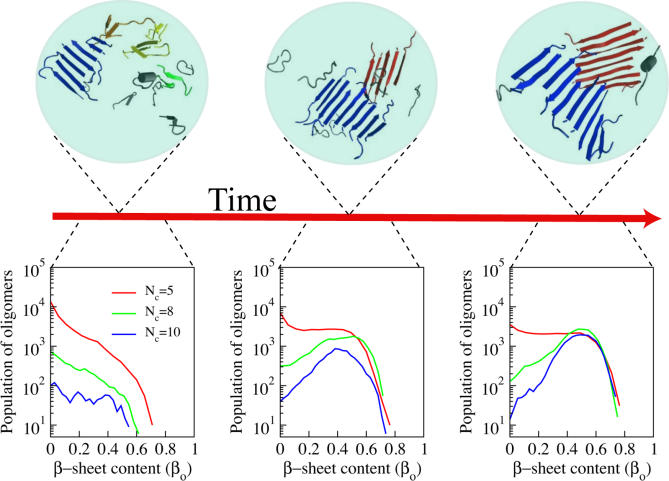
Time Dependence of the Population of Aβ_25–35_ Oligomers The population *P*(*N_c_*) was calculated as the sum over all the oligomers of a given size (*N_c_* = 5 (red), 8 (green), and 10 (blue)) in a specific time window and in all the 100 independent simulations. We show *P* as a function of the β sheet content as defined by the local-order parameter β_o_. Each window corresponds to a different time point along the simulation. At early stages (left), only small ordered oligomers are present, while at intermediate stages (centre), the size of ordered oligomers grows, and at late stages (right), the ordered oligomers are quite large.

Under the conditions used in this study, the aggregation process of the Aβ_16–22_ peptide proceeds without crossing major free energy barriers, so that the lag phase, which is often observed in experimental studies of amyloid formation [6], is suppressed and oligomers start forming immediately without the need for a nucleation event. Thus, from an initial configuration of monomers, we observe the rapid formation of oligomers. These oligomers are themselves transient species and undergo a process of internal reorganisation that can take them through a succession of increasingly ordered states. To analyze the trajectories in a non-equilibrium regime, we divide them into a series of time windows (Figures 2 and 3). We plot the populations *P* of oligomers of given sizes (*N_c_* = 5, 11, and 14 for Aβ_16–22_ in Figure 2, and *N_c_* = 5, 8, and 10 for Aβ_25–35_ in Figure 3) as a function of their degree of local order, *β_o_*.

We observe clear time dependencies both of the oligomer populations and of their degrees of order. For small oligomers (*N_c_* = 5), the data show a decrease in the populations of the disordered species with time. In larger ones, the time evolution is more complicated and so we describe the behavior of the two peptides, Aβ_16–22_ and Aβ_25–35_, separately. For Aβ_16–22_, the population of large oligomers (*N_c_* = 14, Figure 2) does not vary significantly with time but the peak of the *β_o_* distribution shifts toward a higher degree of order. This peptide can therefore be seen to form large disordered oligomers very quickly, which then reorganize and form species with a high content of β sheet structure. Such a mechanism has been described by Serio et al. [14] in order to interpret experimental results for a yeast prion, and experimental evidence that this type of behavior may be general has been reported by Plakoutsi et al. [30], Bader et al. [11] and Petty and Decatur [31]. In the simulations of the Aβ_25–35_ peptide, however, a different scenario emerges as the number of large, predominantly ordered, oligomers (*N_c_* = 10, Figure 3) increases with time. These differences between the time-dependent evolution of large oligomers of Aβ_16–22_ and Aβ_25–35_ indicate an underlying diversity in the fundamental mechanisms of self-assembly, which is analyzed in greater detail in the next section.

**Figure 4:**
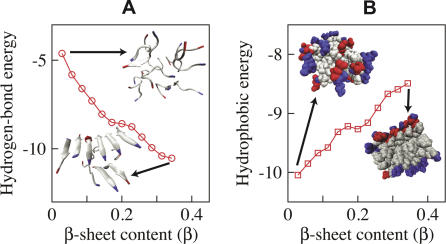
Coalescence and Reorganisation of Aβ_16–22_ Competition between hydrophobicity and hydrogen bonding in the formation of ordered oligomers formed by the Aβ_16–22_ peptide. (A) Average interchain hydrogen-bond energy per chain and (B) average interchain hydrophobic energy with respect to the order parameter β. Two representative oligomer configurations are shown corresponding to β = 0 and β = 0.36; comparison of these structures illustrates the process of internal reorganisation that leads to increases in both the number of hydrogen bonds and in the exposure of hydrophobic groups (gray spheres).

**Figure 5:**
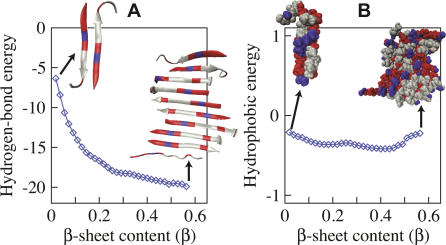
One-Step Ordering of Aβ_25–35_ Oligomers Competition between hydrophobicity and hydrogen bonding in the ordering process of the oligomers formed by the Aβ_25–35_ peptide. As in Figure 4, we report the average interchain hydrogen-bond energy per chain (A), and the average interchain hydrophobic energy with respect to the order parameter β (B). Two representative oligomer configurations are shown corresponding to β = 0 and β = 0.58. As opposed to the case of the Aβ_16–22_ peptide shown in Figure 4, however, there is no evidence of structural reorganization within the oligomers, and therefore even small oligomers are ordered, provided that the overall degree of order in the ensemble, β, is very small due to the large fraction of unstructured monomers present in the system.

Since the analysis of the different populations reveals that oligomers of different sizes undergo a process of reorganisation on different time scales, and hence achieve different degrees of structural order at similar times, it becomes evident that a simple ensemble average, as shown in Figure S1, is not an optimal method of describing the process of aggregation. It is not possible from an ensemble average over the 100 independent simulations to follow the nature of the structural transitions of the oligomers from an analysis of the total hydrogen bond energy, hydrophobic interactions, and β sheet content as a function of time. We observed, however, that the average β sheet content increases over time throughout our simulations, and we, therefore, monitor the average hydrogen bonding and hydrophobic energy for each value of the degree of order β (Figures 4 and 5).

### Coalescence and Reorganisation of Aβ_16–22_

The pathway followed by the Aβ_16–22_ peptides during the process of oligomerization can be divided into two steps. In the first, the monomers present at the beginning of the simulation coalesce rapidly into molten oligomers as a result of strong interactions between hydrophobic residues. The evidence for such a coalescence and its hydrophobic nature is shown in Figure 2, where a population of large oligomers (*N_c_* = 14) is present very early in the simulations, and in Figure 4B, where the initial disordered structures can be seen to be stabilised by strong hydrophobic interactions, as in these early structures the majority of the hydrophobic residues (gray spheres) are buried within the coalesced configurations to form oligomers with very few hydrogen bonding interactions (Figure 4A).

**Figure 6:**
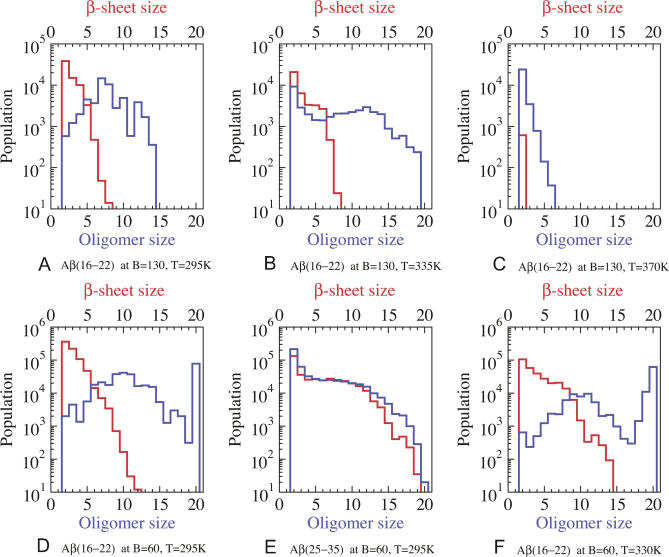
Populations of Oligomers and β Sheets Populations of oligomers (blue) and β sheets (red) as a function of the oligomer and β sheet size for different concentrations and temperatures Aβ_16–22_, box size *B* = 130 and temperature *T* = 295 K (A). (B) Aβ_16–22_, box size *B* = 130 and temperature *T* = 335 K. (C) Aβ_16–22_, box size *B* = 130 and temperature *T* = 370 K. (D) Aβ_16–22_, box size *B* = 60 and temperature *T* = 295 K. (E) Aβ_25–35_, box size *B* = 60 and temperature *T* = 295 K. (F) Aβ_16–22_, box size *B* = 60 and temperature *T* = 330 K.

These oligomers are substantially disordered as the largely nondirectional hydrophobic interactions form rapidly while the hydrogen bonding interactions have a strong distance and angular dependence and therefore form more slowly. In the second step of the assembly process, these collapsed oligomers undergo a process of reorganisation (Figure 2) as revealed by a time-dependent shift toward higher values of the order parameter. This ordering process is driven by the formation of directional interchain hydrogen bonds between the peptides (Figure 4A), which transform the molten oligomers produced by the initial hydrophobic collapse, in which no significant secondary structure is present, into oligomers rich in β sheet structure. As a consequence of this process, many of the hydrophobic residues that are initially buried inside the oligomer become exposed to the solvent (Figure 4B), with a consequent decrease in the contribution of the hydrophobic interactions to the stability of the oligomers. In Figure 6D we show how the population of oligomers and of β sheet structures depends on the sizes of the oligomers and the extent of β sheet structure within them, under the conditions of temperature and concentration used throughout this study. As a consequence of the initial hydrophobic collapse and the subsequent conformational conversion, the two distributions appear very different. This type of reorganisation has been characterised experimentally by Petty and Decatur [31] using isotope-edited infrared spectroscopy and described theoretically as a “reptation” process by Santini et al. [47] using an activation–relaxation computational technique (ART) and by Nguyen et al. [35] with the dock and lock mechanism.

### Direct Ordering of Aβ_25–35_ Oligomers

Aβ_25–35_ is significantly less hydrophobic than Aβ_16–22_. As a consequence of this fact, there is no significant hydrophobic coalescence of molecules leading to the formation of disordered oligomers. Instead, the incorporation of a molecule into an ordered β sheet takes place in a single step, driven by the formation of the interchain hydrogen bonds (Figure 5), such that the oligomers of Aβ_25–35_ grow in a highly ordered fashion. Particularly clear evidence for this type of growth is provided by the data reported in Figure 3, where, even in the first time window, small Aβ_25–35_ oligomers (*N_c_* = 5) are seen to have a relatively high degree of order and to be highly populated, while the number of larger oligomers (*N_c_* = 10) increase significantly more slowly. This behavior is strikingly different from that of Aβ_16–22_ shown in Figure 2, where a substantial population of large oligomers is formed early but the latter become ordered more slowly than do the large oligomers of the Aβ_25–35_ peptide.

**Figure 7:**
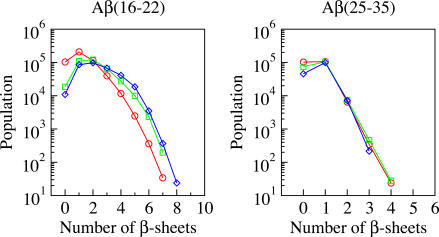
Populations of Oligomers as a Function of the Total Number of β Sheets Contained within the Oligomers The hydrophobic interactions that drive the coalescence and hence the formation of Aβ_16–22_ oligomers (left), results in aggregates with complex structures containing many small β sheets. On the other hand, the hydrogen bond interactions that drive the oligomerization of the Aβ_25–35_ fragments (right) result in simpler structures with a small number of β sheets.

This distinction is also seen from the differences in the nature of the populations of oligomers and β sheets in the simulations of the two peptides. For Aβ_25–35_, as shown in Figure 6E, the two population distributions are almost identical, indicating clearly that the oligomers do indeed grow in an ordered fashion. A further consequence of the differences in the aggregation processes of the two peptides can be seen in Figure 7, where the populations of oligomers are described as a function of the β sheets present within them. The hydrophobic coalescence observed in the formation of oligomers of the Aβ_16–22_ fragment produces complex structures containing several small segments of β sheet, while the more hydrogen bond driven oligomerization of the Aβ_25–35_ fragment results in simple oligomers with a small number of extended β sheet structures.

In addition, the data shown in the last time window in Figure 3 reveal that the distribution of large Aβ_25–35_ oligomers reaches a maximum at high values of the order parameter *β_o_*, while the number of smaller oligomers is effectively the same for a large range of *β_o_* values. In other words, the degree of order of a β sheet increases with its size, a finding that is consistent with the extremely high degree of long-range order found in amyloid fibrils [6,35]. In agreement with our findings, recent studies [54] of a variety of truncated peptides of Aβ_40/42_, the fragment 25–35 of the Aβ peptide was found to form β sheet rich structures more readily than the fragment 17–21. The latter peptide was found, however, to generate much greater toxicity in cellular aggregates than the corresponding fragment 25–35.

### Exposure of Hydrophobic Residues in the Oligomer Species

A series of recent studies has suggested that the toxicity of amyloid aggregates depends on their size [22,25,26], with the maximum toxicity corresponding to those species with amyloid characteristics that have the highest surface-to-volume ratio. In addition, there is increasing evidence of a relationship between the hydrophobicity of the polypeptide chains and the toxicity of the oligomers that they form [27–29]. These results are consistent with the idea that toxicity could result from aberrant interactions with cellular components such as membranes, proteasomes, or molecular chaperones [22,55,56]. Such interactions are likely to be caused by the extended solvent-exposed hydrophobic surfaces exhibited by these oligomers, as probed, for example, by ANS binding experiments [57,58].

**Figure 8:**
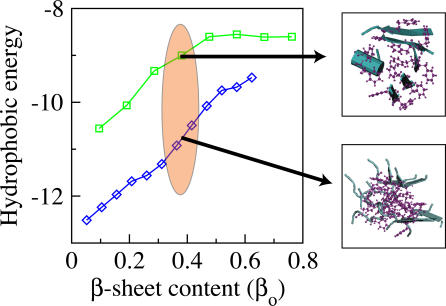
Surface-to-Volume Dependence for the Exposure of Hydrophobic Residues in Oligomeric Species Average hydrophobic energy per peptide for oligomers containing six peptide molecules (i.e., *N_c_* = 6, green line) and 11 peptide molecules (i.e., *N_c_* = 11 blue line) as a function of the β sheet content within the oligomer. As examples, two selected oligomeric configurations, both with the same degree of order, *β_o_* = 0.38, are shown on the right. Large oligomers have a lower surface-to-volume ratio, and this results in a smaller number of exposed hydrophobic residues per peptide molecule.

Recent experiments [54] have suggested that residues 17–21 play a key role in the toxicity of the Aβ_40_ and Aβ_42_ peptides. We therefore studied the time evolution of the solvent-exposed hydrophobic surface of Aβ_16–22_ during the formation of β rich oligomeric assemblies. We exploited the all-atom representation of the Aβ_16–22_ peptides in our simulations to calculate the total number of solvent-exposed hydrophobic residues in the entire ensemble and also the average number per molecule in oligomers of specific sizes (i.e., *S^TOT^* and *S,* respectively, see Methods). Our results show that the nature of the solvent-exposed surface is primarily determined by two competing processes. First, the initially formed collapsed oligomers undergo a conformational transition into β sheet rich structures and expose an increasing number of hydrophobic residues (Figure 4B). Second, the oligomers grow into larger species and their surface-to-volume ratio decreases. The net effect is illustrated in Figure 8, where, as an example, we show two oligomeric configurations with the same relative β sheet content (*β_o_*); smaller oligomers have weaker total hydrophobic interactions and therefore have a larger fraction of exposed hydrophobic residues. These two processes, along with the initial hydrophobic collapse, contribute to *S_n_*(*τ*) in Equation 4.

**Figure 9:**
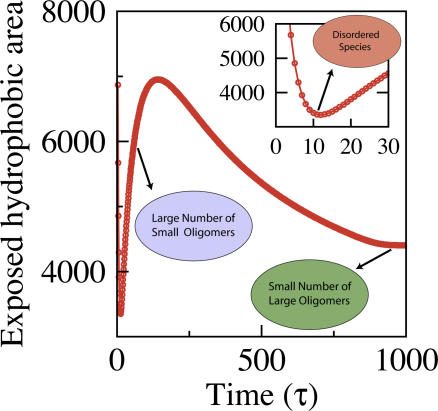
Time Dependence of the Total Exposed Hydrophobic Area in the Oligomeric Ensemble Time dependence of the solvent-exposed hydrophobic surface area (*S*) for the Aβ_16–22_ fragment (see Methods). Two competing processes contribute to the evolution of the species. At early times, *S* increases due to the reorganisation process of the initially formed oligomers driven by the formation of hydrogen bonds. By contrast, *S* decreases as the typical size of the oligomers grows and the surface-to-volume ratio becomes smaller and the consequent decrease in the number of oligomers present in the system. In the inset we show an expansion of the early time points representing the effect of the initial hydrophobic coalescence of the monomers into disordered oligomers.

To describe the process over the whole ensemble of oligomers (*S^TOT^*(*τ*)), however, we need to consider also the distribution of aggregates of different sizes (Equation 3). The result is that the overall number of exposed hydrophobic residues (Figure 9) initially grows due to the increased exposure resulting from the reorganisation of the initially formed oligomers and later decreases due to the combined effect of the decreasing surface-to-volume ratio as the oligomers grow in size and the consequent reduction in the total number of oligomers. Remarkably, this behaviour is at least qualitatively very similar to that of the toxicity reported by Bucciantini et al. [22,26], suggesting that the extent to which hydrophobic residues are exposed could well be a crucial factor in determining the toxic properties of oligomers during the formation of amyloid fibrils and plaques.

## Discussion

**Figure 10:**
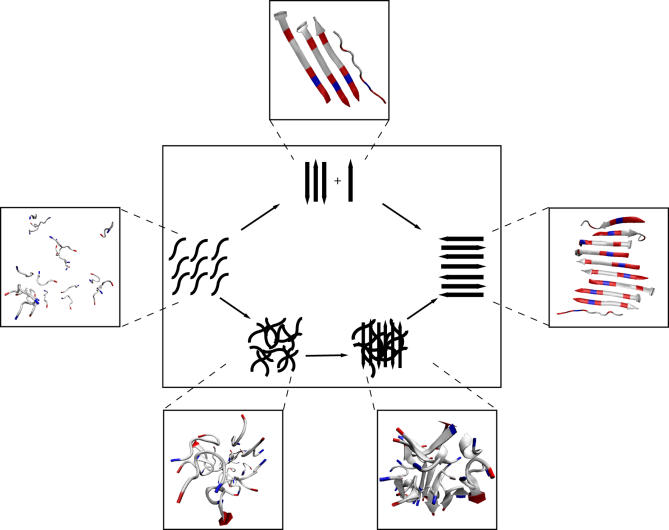
Schematic Diagram of the Two-Step Mechanism of Amyloid Formation Schematic diagram of the mechanistic pathway resulting in the formation of ordered oligomers, described in this study, showing an effective one-step process involving the assembly of monomers directly into β sheet rich oligomers (top) and a more general two-step process, where the monomers coalesce to form molten oligomers before undergoing a process of conformational conversion (bottom). The boxes show representative structures from simulations of Aβ_16–22_ (lower and left panels) and Aβ_25–35_ (top and right panels).

The analysis of the conversion of monomeric Aβ_16–22_ and Aβ_25–35_ peptides into oligomers, observed using computer simulations, suggests that this process occurs by a generic “two-step” mechanism and that it is highly dependent on their degree of hydrophobicity. The more hydrophobic Aβ_16–22_ peptides initially coalesce into disordered oligomers that subsequently undergo a process of conformational conversion that leads to the formation of highly ordered species. By contrast, the hydrophobicity of the Aβ_25–35_ peptides is not sufficient to drive an initial coalescence step, and the peptides instead form ordered aggregates effectively in a single step driven by the formation of hydrogen bonds (Figure 10). This process can be considered to be a special case of the two-step mechanism in which its first step is effectively suppressed.

These results suggest that interchain hydrophobic interactions and the specific sequence of a polypeptide chain are likely to play a major role in promoting the formation of oligomeric species. Indeed, experiments suggest that concentration, temperature, and hydrophobicity all influence the rate of collapse of a given polypeptide chain into different types of aggregates [11]. If the rate of collapse is fast compared with that of conformational conversion, aggregation will take place by a process of coalescence and reorganisation. By contrast, if hydrophobic forces are less important in driving the association of peptide molecules, ordered oligomers essentially assemble directly.

The two-step process that describes amyloid formation is therefore modulated by the competition between hydrogen bonding and hydrophobicity. While the initial coalescence step is driven primarily by the rapid formation of relatively nonspecific hydrophobic interactions, the conformational conversion that results in the formation of ordered β sheet structures is predominantly a consequence of the slow formation of highly directional interchain hydrogen bonds. The fact that this process of conformational conversion has been found both experimentally and theoretically in a range of other studies of a variety of models systems [11,14,30,31,33,35] suggests that this process could indeed be a generic one. Importantly, a mechanism of this type is consistent with the hypothesis that the ability to form fibrils is a common property of polypeptide chains and that the resulting structures have similar structural characteristics regardless of the peptides or proteins involved [4–6]. Experimental evidence for the importance of the competition between hydrogen bonding and hydrophobic interactions in the aggregation process has been found in studies in which changes in the solution conditions that result in a change in the hydrogen bonding interactions led either to fibrillar or nonfibrillar aggregates with similar β sheet content but with distinct morphological features [59].

The results that we have presented in this article provide a detailed description of a process in which the formation of intermolecular hydrogen bonds is at the origin of the generation of β sheets within oligomeric species [31]. By extension, the sequence-independent nature of the hydrogen bonding interactions in oligomers observed in the present study can also rationalize the observation of the inherent toxicity of species formed prior to the mature fibrils. This latter observation has been demonstrated for both disease- and nondisease-related peptides and proteins [22,24,26].

We have also found in the present study that the conformational conversion that results in ordered oligomers also leads to an increase in the number of hydrophobic residues that are exposed to the solvent. This process, however, is offset by the decrease in the surface-to-volume ratio of oligomers as their size increases with time. A combination of these two effects gives rise to the prediction that there will be a maximum in the dependence of toxicity, a phenomenon likely to be related to exposed hydrophobic residues, on aggregate size, and hence on the time over which aggregation occurs. Such a maximum has been observed in experimental studies of the effects of aggregates on the viability of cells in culture and indeed within the brains of higher organisms [22,26]. Our results suggest that the competition between hydrogen bonding and hydrophobic interactions is the crucial factor that modulates the subtle balance between the generic ability and relative propensities of polypeptide molecules to form ordered structures and the potential toxicity of the resulting species to living systems.

## Methods

### Model.

Simulations were carried out with ProFASi [53], which implements an implicit water all-atom model [49–52] for protein folding and aggregation studies. The model assumes fixed bond lengths, bond angles, and peptide torsional angles, so that each amino acid has only the Ramachandran torsional angles and the side chains torsional angles as its degrees of freedom. The interaction potential

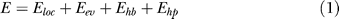
is composed of four terms. The *E_loc_* term is local and represents an electrostatic interaction between adjacent peptides along the chain and the *E_ev_* term is a 1/*r* ^12^ repulsion between pairs of atoms. The hydrogen bonding contributions to the energy are calculated by a term, *E_hb_*, in which the distance dependence is modeled through a Lennard-Jones potential between pairs of NH and CO groups within a given cutoff of 4.5 Å, and the angular dependence is expressed as a function of the *NH*···*O* and *H*···*OC* angles [52]. The hydrophobicity term *E_hp_* is defined by a contact potential between hydrophobic side chains, the latter being proportional to the fraction of atoms in contact in the two amino acids [52]. The parameters of the potential were chosen by optimizing the agreement with the melting temperature of the Trp-cage miniprotein; the resulting force field has been shown to reproduce accurately the folded states and the melting temperatures of a range of polypeptide chains of both α and β structures, including Betanova, GB1p, LLM, and *F_s_*, with excellent agreement with both CD and NMR data [52]. In addition, properties such as the content of α-helix and the relative population of folded species was also found to be in excellent agreement with experimental data [52]. ProFASi has also already been applied to study the aggregation of a series of short peptides, including the Aβ_16–22_ fragment considered here [50]. Those simulations generated a series of conformations of the assemblies of the Aβ_16–22_ peptide containing in register the antiparallel structures, a behaviour previously observed experimentally [60]. The Monte Carlo simulations that we describe in this work were performed at constant temperature in a periodic box of 60 Å using only local updates [61] and rigid body translations and rotations. In Figure 6 we compare the oligomer population and the β sheet population for several such cases. We tested simulations in a cubic box of (130 Å)^3^ at 295, 335, and 370 K and in a cubic box of (60 Å)^3^ at 295 and 330 K. In some cases (Figure 6A and 6C), we do not observe a complete collapse of all the peptides, and in other cases (Figure 6B) the population of oligomers is essentially the same as the β sheet contour. In some systems, see Figure 6F, the oligomer population is artificially skewed toward very large oligomers as a consequence of the finite size of the system. On the basis of these studies, we chose to study the aggregation of both Aβ_16–22_ (Figure 6D) and Aβ_25–35_ (Figure 6E) inside a cubic box with periodic boundary conditions of (60 Å)^3^ and at a temperature of 295 K. We then analysed ensembles of trajectories to identify the major kinetic events during the aggregation process. This type of analysis is based on the observation that if kinetic events are separated by a large number of local moves and are observed in an ensemble of trajectories, they represent significant shifts in population distribution and reflect properties of the free energy landscape such as the depth of local minima and the height of the barriers between them [62].

### The local-order parameter.

To be able to describe the ordering process at the level of individual residues, we introduce the concept of “local order” using the parameter β*_ij_* for *i*-th residue in the *j*-th peptide. To identify the local order (β*_ij_*) of each residue, we follow the procedure of the local-structure vector, ***q*** *_l_*(*i,j*), used by ten Wolde et al. and Auer et al. [63,64], where *l* is the index of the spherical harmonics used to determine the symmetry of local structure near atom *i*. We calculate this vector only for the *C_α_* atoms in each residue. A clear distinction between ordered and disordered residues can conveniently be made by using *l* = 4, since every residue in a β sheet has an approximate fourfold symmetry. The vector ***q*** *_4_*(*i,j*), therefore, reveals the geometrical structure in the vicinity of residue *i* in peptide *j*.

**Figure 11:**
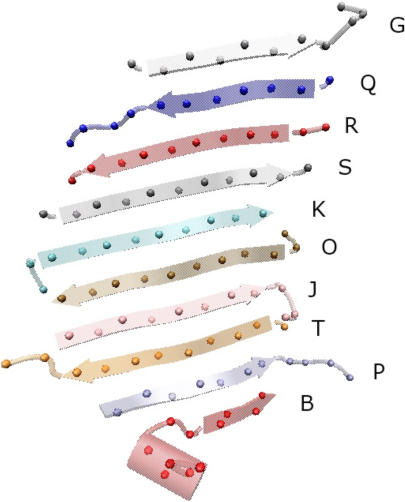
Example of Calculation of the Residue-Specific Local Order An example of an ordered oligomer with *N_c_* = 10 for 20 Aβ_25–35_ peptides after 10^9^ Monte Carlo steps. The spheres on each peptide chain stand for C_α_ atoms. The *β_i,j_* values of each peptide chain are given as G(01111110000), Q(00111111110), R(01111111111), S(01111111110), K(00111111111), O(00111111111), J(01111111000), T(01111110000), P(01111111000), and B(00000000100). The two different ways of averaging the β sheet parameter (β and β_o_) are calculated as β = 111/220 where 111 is the total number of ordered residues in the ensemble of 20 peptides and 220 is the total number of residues (20 × 11), and *β_o_* = 72/110 where 72 is the number of ordered residues in the oligomer shown above and 110 the total number of residues within this particular oligomer (10 × 11).

A high correlation between these vectors calculated over two nearby atoms *i* and *i′* (less than 5.5 Å apart) implies that they have similar local environments [63]. If we find at least three other residues with similar local environments in the vicinity of residue *i*, we define the residue to be ordered and set β*_ij_* = 1, otherwise we define it to be disordered and β*_ij_* = 0. In Figure 11 we calculate the average of the residue-specific local order (β*_ij_*) over the whole ensemble of peptides (β) and over the peptides contained in a single specific oligomer (β*_o_*).

### Measurement of the hydrophobic surface of the oligomers.

By analyzing the results of our simulations, we concluded that the assembly of peptides is described by a Poisson process in which the elementary event consists in the attachment of an oligomer of, on average, 2.2 peptide molecules:

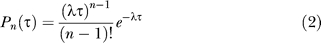



The time (τ) is scaled as τ = t^α^, where α is evaluated from the stretched exponential form of the autocorrelation function of the oligomers. The time dependence of the solvent accessible hydrophobic surface area is calculated as:

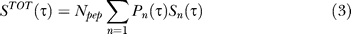



where *N_pep_* is the number of peptides in a system and *P_n_*(τ) is the distribution of the oligomers as in Equation 2. The solvent-exposed hydrophobic surface area per oligomer is expressed as:

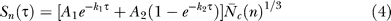



The first term of *S_n_*(τ) represents the coalescence step that typically is driven by the burial of hydrophobic side chains. The second term is the ordering process by which hydrophobic side chains can become exposed. As *N_c_* is proportional to the volume of the oligomer, the term 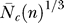 represents their surface-to-volume ratio.

The molecular simulations that we have performed enable the parameters *A_1_*, *A_2_*, *k_1_* , λ, and *k_2_* to be estimated, thus providing a general expression for *S_n_* and *P_n_* for oligomers of any size. We estimated k_2_ = 0.0134, from the ordering of β content in time, and λ = 0.5 by fitting the probability distribution of the oligomers. Values for the other three parameters *A*_1_ = 3.2, *A*_2_ = 9.3, *k*_1_ = 0.207 were obtained from the hydrophobic energy per peptide of oligomers in time.

We use this equation to plot the hydrophobic surface area in Figure 9 for a system of *N_pep_* = 5,000 peptide molecules. The peak time τ = 140 corresponds to 4 × 10^13^ Monte Carlo steps.

## Supporting Information

Figure S1 Time Dependence of the Hydrogen Bond and Hydrophobic Energy and of the β Sheet Content Hydrogen Bond (A), Hydrophobicity (B), and β Sheet Content as a function of time. These quantities are different from the ones shown in Figures 4 and 5. These quantities are calculated as an ensemble average over the 100 independent simulations, while in Figures 4 and 5 the averages are calculated over the ensemble of configurations with the same degree of β sheet content (β). The striking difference between the two behaviours is due to the different time scales of the process of conformational conversion for oligomers of different sizes as shown in Figures 4 and 5.
(958 KB TIF)

## Abbreviations

ProFASi: Protein Folding and Aggregation Simulator
